# Identification of a Novel *NF1* Frameshift Variant in a Chinese Family with Neurofibromatosis Type 1

**DOI:** 10.1155/2019/2721357

**Published:** 2019-12-09

**Authors:** Guoyao Xu, Ming Li, Youya Niu, Xueshuang Huang, Yanchun Li, Genyun Tang, Sha Long, Hui Zhao, Haiou Jiang

**Affiliations:** ^1^Department of Cellular Biology and Genetics, Hunan Provincial Key Laboratory of Dong Medicine, Hunan University of Medicine, Huaihua, Hunan Province, China; ^2^Department of Neurology, The First Affiliated Hospital, Hunan University of Medicine, Huaihua, Hunan Province, China; ^3^Department of Histology and Embryology, Hunan University of Medicine, Huaihua, Hunan Province, China; ^4^Department of Oncology, The First Affiliated Hospital, Hunan University of Medicine, Huaihua, Hunan Province, China

## Abstract

Neurofibromatosis type 1 (*NF1*) is a progressive neurocutaneous disorder in humans, mainly characterized by café-au-lait macules (CALMs) and neurofibromas. *NF1* is caused by variants of the neurofibromin 1 gene (*NF1*), which encodes a Ras-GTPase-activating protein called neurofibromin. *NF1* variants may result in loss of neurofibromin function and elevation of cell proliferation and tumor formation. In this study, a Chinese *NF1* family with an autosomal dominant inheritance pattern was recruited. Exome sequencing and Sanger sequencing were performed to discover the causative variant responsible for the family, followed by molecular analysis of effect of the mutated *NF1* protein on Ras activity. A novel frameshift variant c.541dupC (p.(Gln181Profs*∗*20)) in the *NF1* gene was identified in all three affected family members. The variant cosegregated with the disease phenotypes in the pedigree and was absent in 100 healthy controls. Bioinformatic analysis showed that the variant c.541dupC (p.(Gln181Profs*∗*20)) was pathogenic. The further molecular analysis verified the cells expressing *NF1* variant p.(Gln181Profs*∗*20) partially enhanced Ras activity and elevated cell proliferation and tumor formation due to loss of neurofibromin function caused by the variant. Taken together, the data strongly advocate the c.541dupC (p.(Gln181Profs*∗*20)) variant as the underlying genetic cause of the Chinese family with *NF1*. Moreover, our findings broaden the spectrum of *NF1* variants and provide molecular insights into the pathogenesis of *NF1*.

## 1. Introduction

Neurofibromatosis type 1 (*NF1*; OMIM 162200), also known as von Recklinghausen disease, is a progressive autosomal dominant disorder in humans, mainly characterized by café-au-lait macules (CALMs), neurofibromas, skinfold freckling, and Lisch nodules. *NF1* is one of the most widespread genetic disorders worldwide with a prevalence of 1/3500 live births [1]. Currently, many studies show that *NF1* is solely caused by variants in the neurofibromin 1 gene (*NF1*), which encodes a RAS-GTPase-activating protein called neurofibromin [2, 3]. The *NF1* gene is located on 17q11.2, containing 60 exons and spanning 282,751 bp in length. Loss of neurofibromin function caused by *NF1* variants may lead to enhanced Ras activity and uncontrolled cell proliferation [4]. So far, more than 2700 disease-causing *NF1* variants have been reported in the Human Gene Mutation Database (http://www.hgmd.org), and these variants are distributed throughout the gene [5]. Owing to the large size and complexity of the *NF1* gene, using conventional Sanger sequencing to identify *NF1* variants is extremely time-consuming and expensive; in contrast, exome sequencing is a powerful and cost-effective tool which reveals the genetic basis of the disease [6]. In this study, we first performed a combination of exome sequencing and Sanger sequencing, and the results revealed a novel frameshift variant c.541dupC (NM_001042492.3, p.(Gln181Profs*∗*20)) in the *NF1* gene in a Han Chinese family with *NF1*. Then, molecular analyses demonstrated the cells expressed the *NF1* variant partially enhanced Ras activity and elevated cell proliferation and tumor formation.

## 2. Materials and Methods

### 2.1. Subjects

**Figure 1:**
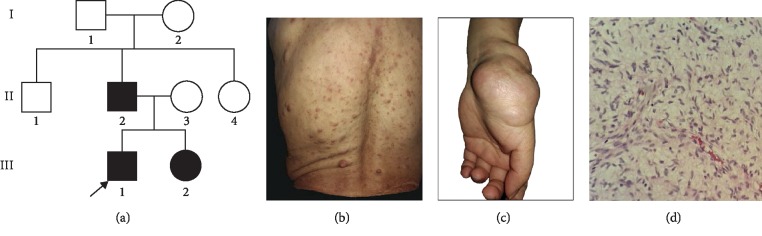
Pedigree and clinical photographs. (a) Affected individuals were indicated by solid squares (males) or circles (females). Normal individuals were indicated by open symbols. Arrow showed the proband. (b) Back of the proband (III1) covered in neurofibromas. (c) A large neurofibroma on the radial side of left hand's thenar of the proband's sister (III2). (d) Biopsy of the proband (III1) showed that spindle-shaped tumor cells with extended wavy nucleus were immersed in a collagen background.

A Han Chinese *NF1* family with autosomal dominant inheritance participated in the study. We obtained written informed consent from all participants and carried out this study according to the Declaration of Helsinki. This study was also approved by the Medical Ethics Committee of Hunan University of Medicine. Eight members (three affected; Figure 1(a)) from the family were enrolled and performed complete dermatological and physical examination. The diagnosis of neurofibromatosis followed the consensus criteria of the National Institutes of Health, while the proband (III1) was diagnosed with *NF1* by excisional biopsy (Figure 1(d)). 100 unrelated ethnically matched normal controls were also recruited in the study for excluding single nucleotide polymorphism (SNP) of the candidate variants.

### 2.2. Exome Sequencing

Genomic DNA (gDNA) was extracted from peripheral blood as described in the manufacturer's instructions (Tiangen Biotech Co. Ltd, Beijing, China). Exome sequencing for the proband was performed by the GENEWIZ-Suzhou, China. According to the manufacturer's protocol, no less than 1.5 *μ*g of genomic DNA was used to construct the exome library. Genomic DNA of the proband was randomly fragmented using Covaris technology, and the DNA library was pooled and hybridized for enrichment of exons using Agilent SureSelect Human All Exon V5. Enriched exome fragments were sequenced on the HiSeq 2000 platform. A mean sequencing depth of 167.42× was obtained to accurately determine variants at 99.44% of the targeted exome. The sequence reads were aligned to a human genome reference obtained from the UCSC database version hg19 (http://genome.ucsc.edu) using the Burrows–Wheeler Alignment tool. SAMtools was used to detect single nucleotide variants (SNVs) and insertions/deletions, and Picard was used to delete duplicate reads (produced mainly during PCR). The strategies of data filtering were as follows: (i) exclude synonymous variants and noncoding region variants; (ii) exclude high-frequency (minor allele frequency >0.01) polymorphisms in the 1000 Genomes Project, dbSNP137, HapMap8, and the YanHuang1 (YH1) project; (iii) extract heterozygous variants; and (iv) extract variants in the known disease-causing gene for *NF1*. To determine the functional consequence of the variants, we first performed functional prediction according to the classification of the variant of the American College of Medical Genetics and Genomics (ACMG) guidelines, including “pathogenic,” “likely pathogenic,” “uncertain significance,” “likely benign,” and “benign.” The software ANNOVAR (Annotate Variation) was used to annotate possible variants.

### 2.3. Verification with Sanger Sequencing

After exome sequencing, Sanger sequencing was used to verify genetic defects. Sequences of primers for potential causative variants in the *NF1* gene (NM_001042492.3) were designed and synthesized as follows: 5′-TCTTTGGGGGAAGAATCTGTTGAA-3′ and 5′-CCTATAGCCACCCTTGAGAGA-3′. PCR was performed with 30 *μ*L reaction mixtures containing 40 ng of genomic DNA, 1.0 *μ*L of the forward and reverse primers for the final concentration 1.0 *μ*M, and 15 *μ*L of 2 × Taq Master Mix (Huiling Biotech Co. Ltd, Shanghai, China). Thermocycling was performed using the following program: initial denaturation at 95°C for 2 min, followed by 35 cycles of 94°C for 10 s, 59°C for 30 s, and 72°C for 1 min, and final extension at 72°C for 5 min. PCR products were purified with the Cycle-Pure Kit (OMEGA; Bio-Tek, Doraville, GA) and sequenced using an ABI PRISM 3730 automated sequencer (Applied Biosystems). Cosegregation analysis was subsequently performed with available DNA samples from family members.

### 2.4. Cell Culture and Transfection

The HEK293T was cultured in DMEM (Gibco, USA) supplemented with 10% fetal bovine serum (FBS, Gibco, USA) and 100 IU/ml penicillin-streptomycin (Sigma, USA). The cells were incubated at 37°C in 5% CO_2_.

The *NF1* expression constructs were generated using human *NF1* cDNA ligated into the BamH 1 and Not 1 sites of the pcDNA3.1 vector. The PCR reaction for *NF1* WT cDNA was performed. The c.541dupC variant was performed with PCR-based mutagenesis. The *NF1* WT plasmid was used as a template, and the site of variant was covered with the internal primers containing Bbs1 recognition sequences. The c.541dupC variant cDNA was ligated to the pcNDA3.1 vector. All primers for constructed plasmids are shown in Table 1.

The plasmid DNA containing *NF1* WT or c.541dupC variant was amplified in DH5*α* and purified using the Plasmid Mini Kit (OMEGA, USA). The constructed *NF1* WT or c.541dupC variant plasmids were validated with sequence analysis. The constructed plasmids were transfected into HEK293T according to the protocol of Lipofectamine® 3000 reagent (Thermo, USA).

### 2.5. Apoptosis and Immunoblotting Analysis

The HEK239T cells were transfected with the *NF1* WT or *NF1* mutant constructed plasmids at 70–80% confluency. After being transfected for 24 h, the cells were harvested and stained with Annexin V-FITC for apoptosis analysis via flow cytometry according to the manual of an Annexin V-FITC Apoptosis Detection Kit (Dojindo, Japan).

After being transfected for 24 h, the cells were collected for immunoblotting. The cell lysates were collected according to our previously published protocol [7]. The protein was separated by 12% SDS-PAGE and transferred onto a PVDF membrane (Millipore, USA). The membrane was incubated with anti-Ras or anti-*β*-actin at 4°C overnight and incubated with anti-rabbit antibodies at room temperature for 1 h. The results of immunoblotting were visualized by chemiluminescence.

### 2.6. Statistical Analysis

All data from 3 independent experiments were represented as the mean ± standard deviation and analyzed with GraphPad Prism 8 and SPSS 24.0 software. The ANOVA was used for analyzing the statistical differences. The *p* < 0.05 was considered as statistically significant.

## 3. Results

### 3.1. Clinical Manifestation

Three patients in the pedigree were clinically diagnosed with neurofibromatosis type 1. The proband (III1) was a 32-year-old man born with CALMs on his back and thighs. He developed skinfold freckling all over the body at the age of 8 years. These skin pigmentation spots increased in number with age. At the age of 12 years, many subcutaneous soft nodules were found on the trunk of the proband, which gradually scattered over his whole body before adulthood. Dermatological examinations revealed nearly 100 subcutaneous neurofibromas in different size over his entire body covering his face, limbs, and particularly the trunk, with a diameter that varied widely from 1 to 3.5 cm (Figure 1(b)). Histopathology of the resected tumor demonstrated a neurofibroma (Figure 1(d)). The father of the proband (II2), a 58-year-old patient, was born with a large CALM on his back. With age, he developed hundreds of subcutaneous neurofibromas and an increased number of CALMs and skinfold freckling all over his body. The younger sister of the proband (III2) was a 29-year-old female with similar clinic manifestation to the proband, but the number of subcutaneous neurofibromas and CALMs was much fewer. At the age of 20, a tender mass was observed on the radial side of her left hand's thenar. This mass was resected twice, but regenerated. The recurrent mass had a diameter of 5.5 cm (Figure 1(c)). Histopathological features of the lesion were in accordance with neurofibroma. Moreover, these patients were obviously shorter than normal. No abnormalities were found on ophthalmologic examination or magnetic resonance imaging (MRI) of the central nervous system in three affected individuals.

### 3.2. Exome Sequencing

**Figure 2:**
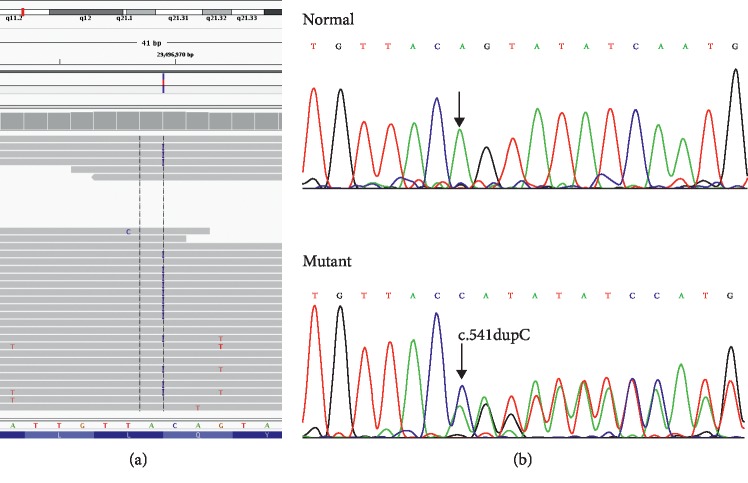
Sequence chromatograms of the variant. (a) Detection of a candidate variant c.541dupC (NM_001042492.3) in exon 5 of *NF1* gene with whole exome sequencing. (b) Validation and cosegregation of *NF1* variant in the family with *NF1*. The frameshift variant c.541dupC (p.(Gln181Profs*∗*20)) was found in all three affected patients and was absent in the normal family members by Sanger sequencing. Sequence chromatograms of the proband (III1) and his healthy mother (II3) were shown. The arrow indicated the mutated site.

The proband generated 103,982,266 raw reads with a mean read length of 150 bp according to exome sequencing; 98.26% (102,172,975) of these raw reads were aligned to the human reference genome. Synonymous variants and known common variants described in dbSNP137, 1000 Genomes data, HapMap8, and the YH1 project were excluded. Nonsynonymous variants were predicted using SIFT, PolyPhen-2, and MutationTaster to eliminate benign variants or tolerated variants. In the known disease-causing gene for *NF1*, a novel heterozygous variant, c.541dupC (NM_001042492.3) and p.(Gln181Profs*∗*20) (NP_001035957.1), in exon 5 of *NF1* gene was identified in the proband (Figure 2(a)). However, no variants of *NF1* modifier genes were found in exome data. We proposed that the slightly phenotypic variability in this family could be due to environmental factors.

### 3.3. Identification of Causative Variants

**Figure 3:**
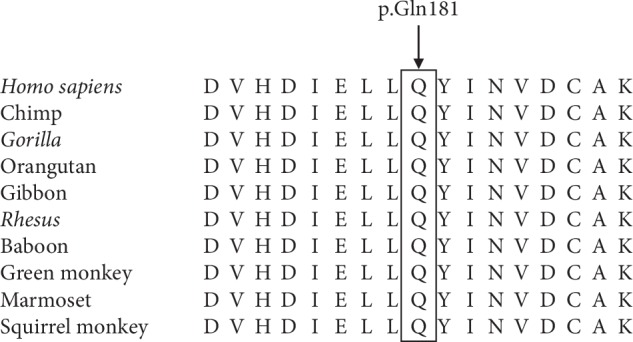
Multiple sequence alignment of *NF1* from different species. The results indicated high conservation of the amino acid sequence around the Gln181 residue across 10 different species.

The heterozygous variant c.541dupC (p.(Gln181Profs*∗*20)) in the *NF1* gene (Figure 2(b)) was found in all three affected family members according to Sanger sequencing and was absent in the healthy family members and 100 ethnically matched normal controls. The variant in the *NF1* gene cosegregated with the disease phenotype in this family, suggesting that the c.541dupC (p.(Gln181Profs*∗*20)) variant was likely responsible for the *NF1* in this family. Multiple sequence alignment across 10 different species indicated a high degree of conservation around the Gln181 residue of the neurofibromin (Figure 3). The variant of the *NF1* gene was predicted to be likely pathogenic according to ACMG, and functional prediction by MutationTaster revealed that this variant was also disease-causing. Besides, the c.541dupC was novel since it had not been previously reported nor was it present in dbSNP, HGMD, or Exome Variant Server.

### 3.4. The p.(Gln181Profs*∗*20) Variant Partly Abolished *NF1*-Induced Apoptosis

**Figure 4:**
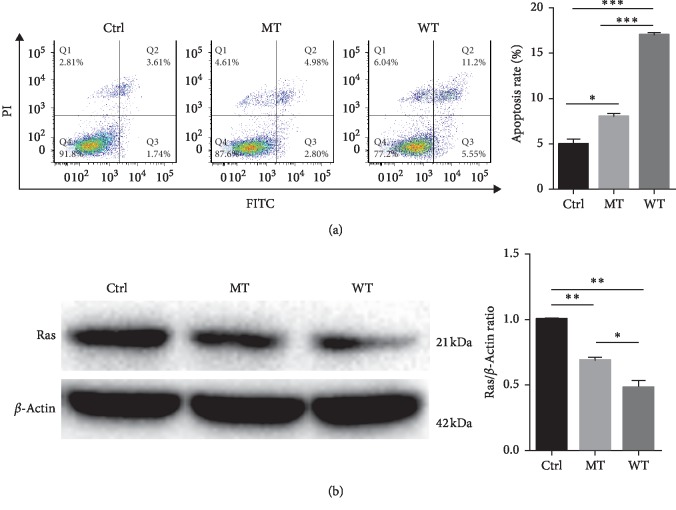
Apoptosis and western blotting measures. The apoptosis (a) and western blotting (b) were analyzed in HEK293T cells overexpressed control or *NF1* p.(Gln181Profs*∗*20) variant or *NF1* WT. All data from 3 independent experiments were represented as the mean ± standard deviation (two-way ANOVA, ^*∗*^*p* < 0.05, ^*∗∗*^*p* < 0.01, and ^*∗∗∗*^*p* < 0.001). The results revealed the p.(Gln181Profs*∗*20) variant partly abolished *NF1*-induced apoptosis.

After being transfected for 24 h, the cells were harvested for apoptosis analysis. The results are shown in Figure 4(a), and the apoptotic rate of the cells with overexpressed *NF1* WT was 16.7%, which was apparently higher than the cells with control vector (Ctrl) (*p* < 0.001). Meanwhile, the apoptotic rate of the cells with overexpressed *NF1* p.(Gln181Profs*∗*20) mutant (MT) was higher than that of the control group (*p* < 0.05), whereas the apoptotic rate of MT was significantly lower than that of the WT group (*p* < 0.001).

To delineate the molecular mechanism of *NF1* MT in apoptosis, the western blotting was performed for monitoring expression of Ras regulated by *NF1*. As shown in Figure 4(b), the expression of Ras in the cells with overexpressed *NF1* was significantly lower than that in the control cells (*p* < 0.01). However, cells overexpressed with *NF1* MT were higher than with *NF1* WT (*p* < 0.05), but markedly lower than that of control cells (*p* < 0.01), indicating *NF1* MT attenuated capacity of *NF1* downregulated Ras expression.

Taken together, the results demonstrate that *NF1* p.(Gln181Profs*∗*20) mutant partly abolished *NF1*-mediated apoptosis via inhibiting downregulated Ras expression.

## 4. Discussion

Neurofibromatosis type 1 is a rare neurocutaneous genetic disease with two major clinical symptoms, i.e., neurofibromata and the café-au-lait spots. The significant advances in the understanding of *NF1* etiology are attributed to the discovery of the *NF1* gene. Neurofibromin encoded by *NF1* gene is a large multidomain protein consisting of 2818 amino acids, which contains a RAS-GTPase-activating protein-related domain that can inactivate p21-RAS by converting the active p21-RAS-GTP to the inactive p21-RAS-GDP [3, 8, 9]. RAS is a crucial component in the RAS-MAPK signaling pathway, in which neurofibromin functions as a regulator of signals for cell proliferation and differentiation, while being short of neurofibromin, it will promote uncontrolled cell proliferation [10]. Therefore, neurofibromin is thought to act as a tumor suppressor. Variants in the *NF1* gene lead to a loss in neurofibromin function, causing downstream cell growth activation [11, 12]. Up to now, many types of variants have been reported, including chromosome abnormalities, base substitutions, insertions, deletions, splice-site variants, 3′-untranslated region variants, and frameshift variants. However, no true variant hot spots have been found in *NF1*, and variants identified so far are randomly scattered within the *NF1* gene [13]. In this Chinese pedigree, a novel frameshift variant c.541dupC (p.(Gln181Profs*∗*20)) in exon 5 identified brings about a premature stop codon at codon 200, which results in a truncated protein of 199-amino acid residues instead of full-length neurofibromin. This produces a variant neurofibromin which loses the entire functional domains. The novel variant is a loss of function mutation in the light of the classification guidelines of ACMG. Moreover, multiple sequence alignment from 10 different species reveals a high degree of conservation around the Gln181 residue of the neurofibromin, demonstrating its functional importance and the potential pathogenicity of the variant. The majority of disease-causing *NF1* variants are truncating variants, which are predicted to generate haploinsufficiency of the neurofibromin owing to the nonsense-mediated mRNA decay (NMD), the shortage of functional domains, or the degradation of the truncated proteins [14]. This might interpret the occurrence of *NF1* in the Chinese family owing to the c.541dupC frameshift variant of *NF1* gene.

In addition, molecular mechanism of c.541dupC (p.(Gln181Profs*∗*20)) frameshift variant of *NF1* gene identified in the Chinese family was further studied. The results revealed that this variant partly abolished the function of *NF1* protein, which might lead to increased activation of the RAS-MAPK pathway. It was likely that the RAS-GTPase activation domain was lost or this domain was unexposed due to the *NF1* truncation variant [15, 16]. Therefore, the activity of RAS was not completely inhibited by the hydrolysis of the GTP; subsequently, the RAS-MAPK signaling pathway was partially activated and thereby promoted uncontrolled cell proliferation and tumor formation for the Chinese family.

## 5. Conclusion

In conclusion, by a combination of exome sequencing and Sanger sequencing, we found a novel disease-causing variant (c.541dupC) in the *NF1* gene from a Chinese family with *NF1*. Functional research implied that this novel variant may enhance Ras activity and elevate cell proliferation and tumor formation. The current study expands the spectrum of *NF1* variants and provides further evidence that the loss or decreased function of the neurofibromin results in *NF1*. In addition, whole exome sequencing can be used for exact and rapid identification of *NF1* variants to establish the molecular diagnosis of *NF1*.

## Figures and Tables

**Table 1:** The primer of *NF1* for plasmids constructed.

Names of primer	Nucleotide sequence (5′-3′)
*NF1* F	GATCGGATCCATGGCCGCGCACAGGCCG
*NF1* R	GATCGCGGCCGCTCAAGACAAAAATACAAA
*NF1* c.541dupC F	GATCGGATCCATGGCCGCGCACAGGCCG
*NF1* c.541dupC Fm	GAAGACCTAATTGTTACCAGTATATCA
*NF1* c.541dupC Rm	GAAGACCTAATTCTATATCATGAACA
*NF1* c.541dupC R	GATCGCGGCCGCTCAAGACAAAAATACAAA

## Data Availability

The data used to support the findings of this study are included within the article.

## References

[1] Shofty B., Constantini S., Ben-Shachar S. (2015). Advances in molecular diagnosis of neurofibromatosis type 1. *Seminars in Pediatric Neurology*.

[2] Xu W., Yang X., Hu X., Li S. (2014). Fifty-four novel mutations in the *NF1* gene and integrated analyses of the mutations that modulate splicing. *International Journal of Molecular Medicine*.

[3] Cai S. P., Fan N., Chen J. (2014). A novel *NF1* frame-shift mutation (c.702_703delGT) in a Chinese family with neurofibromatosis type 1. *Genetics and Molecular Research*.

[4] Schubbert S., Shannon K., Bollag G. (2007). Hyperactive Ras in developmental disorders and cancer. *Nature Reviews Cancer*.

[5] Zhang J., Tong H., Fu X. (2015). Molecular characterization of *NF1* and neurofibromatosis type 1 genotype-phenotype correlations in a Chinese population. *Scientific Reports*.

[6] Stella A., Lastella P., Loconte D. (2018). Accurate classification of *NF1* gene variants in 84 Italian patients with neurofibromatosis type 1. *Genes*.

[7] Li M., Wu X. M., Gao J. (2018). Mutations in the P10 region of procaspase-8 lead to chemotherapy resistance in acute myeloid leukemia by impairing procaspase-8 dimerization. *Cell Death & Disease*.

[8] Xu G., Lin B., Tanaka K. (1990). The catalytic domain of the neurofibromatosis type 1 gene product stimulates ras GTPase and complements ira mutants of S. cerevisiae. *Cell*.

[9] Ratner N., Miller S. J. (2015). A RASopathy gene commonly mutated in cancer: the neurofibromatosis type 1 tumour suppressor. *Nature Reviews Cancer*.

[10] Viskochil D., Friedman J. M., Gutmann D. H., MacCollin M., Riccardi V. M. (1999). The structure and function of the *NF1* gene: molecular pathophysiology. *Neurofibromatosis: Phenotype, Natural History, and Pathogenesism*.

[11] Hirbe A. C., Gutmann D. H. (2014). Neurofibromatosis type 1: a multidisciplinary approach to care. *The Lancet Neurology*.

[12] Arun D., Gutmann D. H. (2004). Recent advances in neurofibromatosis type 1. *Current Opinion in Neurology*.

[13] Rasmussen S. A., Friedman J. M. (2000). *NF1* gene and neurofibromatosis 1. *American Journal of Epidemiology*.

[14] Ars E., Kruyer H., Morell M. (2003). Recurrent mutations in the *NF1* gene are common among neurofibromatosis type 1 patients. *Journal of Medical Genetics*.

[15] Gos M., Leszkiewicz M., Abramowicz A. (2012). RAS/MAPK signal transduction pathway and its role in the pathogenesis of Noonan syndrome. *Postepy Biochemii*.

[16] Jett K., Friedman J. M. (2010). Clinical and genetic aspects of neurofibromatosis 1. *Genetics in Medicine*.

